# An experimentally informed computational model of neurovestibular adaptation to altered gravity

**DOI:** 10.1113/EP091817

**Published:** 2024-04-16

**Authors:** Victoria G. Kravets, Torin K. Clark

**Affiliations:** ^1^ Ann and H.J. Smead Department of Aerospace Engineering Sciences University of Colorado Boulder Colorado USA

**Keywords:** astronaut, Bayesian inference, centrifugation, hypergravity, sensorimotor, spaceflight

## Abstract

Transitions to altered gravity environments result in acute sensorimotor impairment for astronauts, leading to serious mission and safety risks in the crucial first moments in a new setting. Our understanding of the time course and severity of impairment in the early stages of adaptation remains limited and confounded by unmonitored head movements, which are likely to impact the rate of adaptation. Here, we aimed to address this gap by using a human centrifuge to simulate the first hour of hypergravity (1.5*g*) exposure and the subsequent 1*g* readaptation period, with precisely controlled head tilt activity. We quantified head tilt overestimation via subjective visual vertical and found ∼30% tilt overestimation that did not decrease over the course of 1 h of exposure to the simulated gravity environment. These findings extended the floor of the vestibular adaptation window (with controlled vestibular cueing) to 1 h of exposure to altered gravity. We then used the empirical data to inform a computational model of neurovestibular adaptation to changes in the magnitude of gravity, which can offer insight into the adaptation process and, with further tuning, can be used to predict the temporal dynamics of vestibular‐mediated misperceptions in altered gravity.

## Abstract (highlights)

- **What is the central question of this study?**
  Early progression of vestibular impairment during adaptation to altered gravity is poorly characterized. Tilt misperception during the first hour of adaptation, with experimenter‐controlled and well‐quantified orientation cues, has not been assessed previously, and existing computational models of neurovestibular adaptation to altered gravity are not empirically informed.
- **What is the main finding and its importance?**
  Subjects exhibited ∼30% tilt overestimation in 1.5*g*, which did not decay over 1 h of centrifugation. These findings extend the floor of the vestibular adaptation window and bound parameters of a model of neurovestibular adaptation to altered gravity.

## 1 INTRODUCTION

Space exploration exposes astronauts to a variety of safety and health risks, including acute sensorimotor impairment resulting from transitions to an altered gravity environment. Symptoms of sensorimotor impairment have affected every interviewed crew member (Bloomberg et al., 2015; Clark, 2019), resulting in severe discomfort at best and life‐threatening risks at worst (e.g., in the case of mistakes during emergency egress or incorrect pilot inputs during landing owing to spatial disorientation) (Braithwaite et al., 1997; Poisson & Miller, 2014). These symptoms include space motion sickness (Lackner & DiZio, 2006), disorientation (Paloski et al., 2008), inhibited posture (Wood et al., 2015) and hindered locomotion (Mulavara et al., 2018).

Tilt perception is a quantifiable metric of neurovestibular response to changes in the magnitude of gravity, which often presents in the form of a tilt‐gain, or ‘G‐excess’, illusion. The ‘G‐excess’ illusion describes the tendency for individuals to perceive roll tilt as larger than the actual tilt when in a hypergravity environment owing to increased utricular shear stimulation (Clark, 2019; Schöne, 1964). For example, upon return to 1*g* after extended exposure to microgravity, astronauts overestimate their tilt for up to 1–2 days (measured by their perception of a centrifugation‐induced roll‐tilt sensation without canal cues) (Clément & Wood, 2014). The initial altered gravity‐driven tilt misperceptions have also been re‐created in a laboratory setting using human‐rated centrifuges, because subjects experience the summed gravitational and centripetal acceleration, or net gravito‐inertial force (GIF), as the direction and magnitude of the ‘gravitational’ vertical. Tilt perception within the context of the altered GIF environment can then be measured. Centrifuges have been used to explore roll‐tilt perception in simulated hypergravity (i.e., >1*g*) (Clark et al., 2015), and centrifuges or parabolic flights have also been used to create hypogravity (i.e., <1*g*) environments (Clark & Young, 2017; Galvan‐Garza et al., 2018; Meskers et al., 2021). These studies and additional evidence from aircraft experiences lead to an abundance of evidence of altered gravity‐related tilt misperception in the form of overestimation of tilt in hypergravity (Clark et al., 2015; Miller & Graybiel, 1966; Schöne, 1964) and underestimation of tilt in hypogravity (Clark & Young, 2017; Galvan‐Garza et al., 2018; Meskers et al., 2021).

Over the course of extended exposure to altered gravity, tilt misperceptions and other sensorimotor impairments subside, as the brain relearns how to process and interpret incoming sensory information accurately within the context of the new GIF environment. This adaptive process enables individuals to regain stability and perform tasks effectively (Shelhamer, 2015). However, we have not been able to observe extended durations of the adaptation process in a controlled laboratory setting (i.e., with controlled/monitored head and body movements from the onset of altered gravity exposure, which we anticipate will have a substantial impact on the time course of adaptation), and thus have limited understanding of the time course and severity of neurovestibular impairment during the adaptation trajectory. For example, Clark et al. (2015) found less overestimation in a second 15 min hypergravity session on the same day, but the activities of subjects between sessions were not monitored.

The perception of orientation in both normal and altered gravitational environments typically incorporates sensory information from multiple sensory systems, such as vestibular, visual and proprioception (Clark, 2019; Howard, 1982; Newman, 2009). Here, we focus on vestibular contributions to this complex process. The vestibular system is composed of a set of organs in the inner ears. The otoliths sense linear acceleration and gravity, and the semicircular canals sense angular velocity. In humans, these noisy sensors transduce the actual orientation of the body to yield sensory afference (measurements) while incorporating sensory dynamics [e.g., human semicircular canals have high‐pass filter characteristics that lead to relatively poor transduction of low‐frequency rotations compared with high‐frequency rotations (Goldberg & Fernandez, 1971; Haque et al., 2004)].

The CNS is believed to compute expectancy sensory afference using internal models of sensory dynamics, or neural systems that replicate the dynamics of physical systems (Merfeld & Zupan, 2002; Merfeld et al., 1999; Tin & Poon, 2005), which incorporate an assumption of the magnitude of gravity. It has been hypothesized that disparities between actual and expected afference leads to ‘sensory conflict’ signals, driving dynamic updates of states in the internal model (Brooks et al., 2015; Mackrous et al., 2019). Errors in the assumption of the magnitude of gravity by the internal model generate sensory conflict between expected afference and actual incoming sensory signals, compelling an adaptation of the central hypothesis of gravity.

As we aim to explore vestibular adaptation to altered gravity, computational models offer the capacity to simulate and investigate the internal neural computations involved in the adaptation process based on behavioural measurements, even in the absence of direct access to the neural processes. The perception of orientation in static 1*g* environments has been modelled using the well‐validated ‘observer’ model of spatial orientation perception (Merfeld & Zupan, 2002; Merfeld, Young, Oman, et al., 1993; Merfeld, Young, Paige, et al., 1993; Vingerhoets et al., 2006, 2007), which has been updated to model misperceptions in a static hypergravity environment where the subject remains adapted to 1*g* (Clark et al., 2015). Leveraging ‘observer’ as the base model of orientation perception, we have previously proposed the COMPASS (computations for orientation perception in altered sensorimotor states) model of vestibular adaptation to altered environments (Kravets et al., 2021) and have suggested updates to the model incorporating dynamically learned hypotheses of gravity (Kravets et al., 2022) and memory of previously learned gravitational states (Allred et al., 2023). In summary, this family of models captures the neural computations involved in the adaptation process to a new gravity level over time, using alternative hypotheses for the internal assumption of the magnitude of gravity, computing sensory conflict signals for each, and applying Bayesian inference to update the assumed magnitude of gravity. However, the proposed model framework previously lacked the experimental evidence of vestibular adaptation time lines necessary to tune and validate the model.

Here, we explore experimentally the effect of changes in the magnitude of gravity on vestibular‐mediated tilt perception during the first hour of the adaptation period. We then use the collected data to inform the COMPASS model of vestibular adaptation, suggesting possible tuning parameters that can be refined further with future experimentation.

## 2 MATERIALS AND METHODS

### 2.1 Ethical approval

The experimental protocol conformed to the standards set by the *Declaration of Helsinki*, except for registration in a database, and was approved by the Institutional Review Board at the University of Colorado‐Boulder (protocol #22‐0651). Following a thorough verbal and written explanation of the nature and possible risks of the study, volunteer participants provided written informed consent.

### 2.2 Subjects

Thirteen adults (six females and seven males), with a mean ± SD age of 26.8 ± 6.1 years, participated in all segments of the study. One additional subject withdrew from participation during the first few minutes of hypergravity exposure on the first day of testing owing to motion sickness and is thus excluded from analysis. Inclusion criteria required subjects to be between 18 and 50 years of age and to have no history of vestibular dysfunction. Although subjects were not included or excluded based on susceptibility to motion sickness, all subjects completed the motion sickness susceptibility questionnaire (MSSQ) (Golding, 1998, 2006) and scored, on average, in the 24th (±16 SD) percentile. Before the experimental sessions, participants refrained from consuming alcohol for 12 h, and no subjects reported experience with an altered gravity environment (e.g., centrifugation experience) within the month before testing.

### 2.3 Experimental procedure

#### 2.3.1 Altered gravity protocol

**FIGURE 1:**
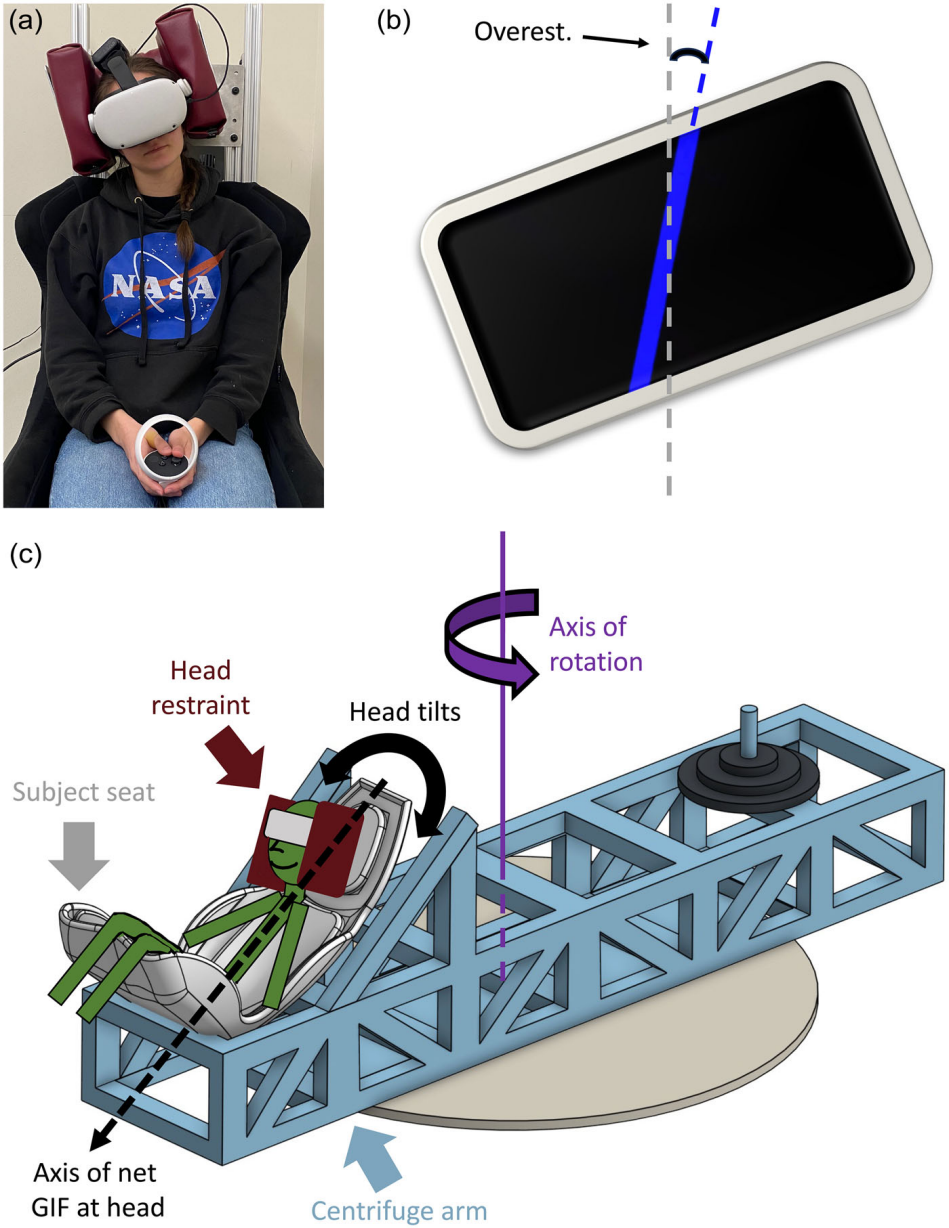
Experimental set‐up. (a) The subject was seated with their head secured and donned a Meta Quest 2 head‐mounted display. Every 2–3 min, their head was tilted passively to a roll tilt angle between 10° and 20°. They held a hand controller with a joystick that allowed them to control the angle of subjective visual vertical (SVV) lines. (b) A see‐through image of the head‐mounted display that an individual in the position shown in panel (a) might see during an SVV trial, from the perspective of the reader. Any error between true vertical and the final line position was considered tilt overestimation (positive overestimation if the line was positioned past vertical; negative overestimation, or underestimation, if it was positioned on the head side of vertical). (c) During the hypergravity segment, the subject was seated in a backward‐pitched orientation, facing towards the ceiling while the centrifuge was stationary. When the centrifuge was at its maximal sustained speed (25 r.p.m.), the subject felt as if they were seated upright, and the net gravito‐inertial force (GIF) (at the centrifuge radius at the head) was along their longitudinal body axis. Identical to the baseline segment set‐up, the subject's head was restrained in a motorized tilt mechanism that generated preprogrammed, operator‐controlled roll tilts (about the naso‐occipital axis) relative to their body axis (and the net GIF direction). All three panels show an example of a rightward roll tilt.

The experimental procedures consisted of three data collection segments repeated on 2 days consecutively: a ‘Baseline’ segment in 1*g* lasting ∼10 min; a ‘Hypergravity’ (1.5*g*) centrifugation segment nominally lasting 1 h; and a ‘1*g* Readaptation’ segment immediately post‐centrifugation lasting 1 h (although it took, on average, 12.8 min to slow down the centrifuge safely and position subjects before beginning post‐centrifugation data collection). In all experimental segments, subjects donned a Meta Quest 2 head‐mounted display (HMD), and their heads were secured with their naso‐occipital axis perpendicular to their longitudinal body axis and the net GIF between roll tilts (i.e., 0° pitch) in a head restraint capable of generating preprogrammed and operator‐controlled roll tilts while limiting uncontrolled head motion (Figure 1a). A customized short‐radius laboratory centrifuge, the Human Eccentric Rotator Device (HERD; Gyrostim, Colorado Springs, CO, USA) located in the University of Colorado Boulder Bioastronautics Laboratory, was used to generate a net GIF equal to 1.5 times Earth gravity, at the location of the centre of their head, along the longitudinal body axis of the subject when at the maximum centripetal acceleration (Figure 1c). The motion device included a head restraint/tilt mechanism set‐up identical to the upright set‐up used for Baseline and 1*g* Readaptation segments. Subjects’ heads were positioned between 1.55 and 1.65 m from the centre of rotation (depending on subject height, the seat and head restraint were adjustable to ensure that the head positioning was in this range), and their feet were positioned at ∼2.9 m from the centre of rotation. To achieve 1.5*g* GIF at the centre of the head, the centrifuge spin rate was 25 r.p.m.

#### 2.3.2 Tilt perception trials

During each of the experimental segments, subjects completed a series of tilt perception trials, involving a head tilt and a series of tilt perception measures. For a single trial, the subjects’ heads were tilted passively relative to their body (which remained untilted) to a random roll angle (about the naso‐occipital axis) between 10° and 20° in either the left or right directions, using a motor attached to the head restraint. The tilt rate was limited to 1°/s to minimize both cross‐coupled Coriolis effects [which could occur in the Hypergravity segment owing to the centrifugation spin (Bretl & Clark, 2020)] and effects of semicircular canal influence on tilt perception.

Once at a static tilt (i.e., at a random roll tilt angle between 10° and 20°), the tilt perception of subjects was measured using subjective visual vertical (SVV) tasks (Tarnutzer et al., 2009), during which the subjects were instructed to orient a virtual blue line (Figure 1b), projected in the HMD, to what they perceived as vertical/gravitational down using a joystick on a hand controller. A series of five SVV replicates were collected during every head‐on‐body tilt (i.e., tilt perception ‘trial’), each with a time limit of 12 s, for a maximum of 1 min at the static head tilt. After the fifth SVV replicate, the head was rotated back to centre at a rate of 1°/s. Subjects reported verbally how many degrees of tilt they had felt while statically tilted and in which direction. Verbal reports were given while subjects were returning to upright.

During Baseline data collection, tilt perception trials occurred every 2 min, and during Hypergravity and 1*g* Readaptation segments, they occurred every 3 min, leading to six tilts during the Baseline segment and 21 tilts during both the Hypergravity and 1*g* Readaptation segments. Between tilt perception trials during the Baseline and Hypergravity segments, subjects were seated with their head aligned with their body, and the HMD showed a black screen.

Subjects were given three training tilt perception trials before Baseline data collection on the first day of testing (without feedback on the accuracy of tilt reporting) to familiarize them with the tasks. To confirm that there was no change in SVV tilt reporting owing to task learning, we used a Wilcoxon signed rank test to compare the mean Baseline tilt perception reports by subjects on day 1 and day 2 of testing and found no significant difference between consecutive days of testing (*W* = 26; *P *= 0.191), suggesting that there was no significant task familiarization effect.

#### 2.3.3 Vestibular orientation cueing

Computational modelling results have suggested that more dynamic head tilts would increase the adaptation rate (Kravets et al., 2021). To explore this hypothesis, we introduced additional motion stimuli during the 1*g* Readaptation segment for a subset of the subjects. Although all subjects experienced identical vestibular cueing (resulting from tilt perception trial head tilts) during the Baseline and Hypergravity segments, 6 of the 13 subjects experienced an additional, controlled 1°/s 10° head dynamic tilt between each tilt perception trial (while the HMD showed a black screen) during only the 1*g* Readaptation segment. As will be seen in the Results, we saw no meaningful differences between these six subjects and the other seven in the first 15 min of the 1*g* Readaptation segment when adaptation‐related effects were most plausible, hence for our primary analyses, all 13 subjects were pooled together.

#### 2.3.4 Motion sickness reporting

Subjects had continuous two‐way audio/verbal communication with the operators throughout the experiment. In addition to reporting verbally how many degrees of tilt they felt, they also rated their motion sickness on a scale of 0–10 using the misery index scale (MISC) (Bos et al., 2005) after each tilt perception trial. Based on subject comfort and operator discretion, centrifugation could be concluded before the 1 h mark, although subjects were still allowed to continue with the 1*g* Readaptation data collection even if they concluded the Hypergravity segment prematurely.

### 2.4 Analysis

#### 2.4.1 Data processing and hypothesis testing

We visualized the sequence of five SVV reports that occurred during each head tilt (as they occurred over time while the head was statically tilted) and found no consistent temporal effects. Thus, the five reports were considered replicates, and we took the median to produce a single SVV tilt perception metric for each head tilt that remained robust to outlier reports. Replicates in which subjects reported verbally that they accidentally mis‐clicked the hand controller were excluded from analysis. Additionally, if subjects had confirmed the placement of the SVV line <0.2 s after the line appeared, the selection was assumed to be a mis‐click and was excluded from analysis. Of the ∼5500 total SVV replicates administered, 68 (1.2%) were either missed reports (i.e., the subject did not confirm a line selection within 12 s) or excluded as mis‐clicks.

Appropriate tilt perception measures were dependent on the head being aligned with the head restraint and on the HMD being aligned on the subject's head between SVV trials, because the line orientation was presented and measured in the HMD reference frame. We compensated for this possibility of left/right roll misalignment or shift in alignment during the experimental segment by adjusting the SVV reported tilt perceptions in our data analysis. This left/right adjustment was subtracted from the raw measured SVV tilt to yield an adjusted metric of SVV tilt perception:

$$\;{\hat{\mathrm{tilt}}}_{\mathrm{adj}}={\hat{\mathrm{tilt}}}_{\mathrm{raw}}-\left[\mathrm{mean}\left({\hat{\mathrm{tilt}}}_{\mathrm{raw}}\right)-\mathrm{mean}\left(\mathrm{til}t_{\mathrm{act}}\right)\right],$$

where $\hat{\mathrm{tilt}}$ is the tilt perception across an experimental segment (left/right adjusted or raw) and $\mathrm{til}t_{\mathrm{act}}$ is the actual tilt angle in the corresponding trial. To account for the possibility of a major head shift during an experimental segment, a sliding time window split the segment into two (not necessarily equal) parts, and a left/right adjustment was calculated for the front and back portions at each possible time point, normalized by the mean left/right adjustment during that segment. If there was a time point at which the difference between the front and back normalized adjustment values exceeded a threshold of two (i.e., there was an apparent, substantial head shift at that point), a different adjustment would be applied to the two portions of the experimental segment. We emphasize that this adjustment could impact only the left/right bias in SVV tilt perception reporting and could not account for any change in the ‘gain’ of SVV reports that would correspond to overestimation of tilt, which are of primary interest.

Next, for each subject, we computed the mean of the six Baseline tilt perceptions, each divided by the actual tilt in each of those six trials, which we refer to as the ‘baseline gain’. A baseline gain value of one would correspond to tilt perception similar to actual tilt, but some subjects, even in the Baseline segment, would report perceptions larger or smaller than the actual tilt angle. To isolate the effect of the Hypergravity segment, we adjusted for the ‘baseline gain’ of each subject in computing the ‘Overestimation’, as follows:

$$\mathrm{Overestimation}=\left[\frac{{\hat{\mathrm{tilt}}}_{\mathrm{adj}}}{\mathrm{baseline}\;\mathrm{gain}}-\mathrm{til}t_{\mathrm{act}}\right]\times \mathrm{sign}\left(\mathrm{til}t_{\mathrm{act}}\right).$$

Overestimation is thought to be a function of actual tilt (Clark et al., 2015; Miller & Graybiel, 1966; Schöne, 1964) and is thus often reported in terms of overestimation gain. However, here the actual tilt angles were all in a 10° range and randomized; therefore, we primarily report tilt overestimation in degrees for ease of interpretation.

When analysing tilt overestimation in the 1*g* Readaptation segment, we included only the first five tilt perception trials (i.e., 12 min), because we wanted to isolate any effects of adaptation to hypergravity and suspect that these effects diminish quickly once back in a 1*g* environment. The remaining 48 min of the 1*g* Readaptation period are visualized, though not analysed, in Results.

Once the data were processed, the effect of testing day and time (in minutes, where each trial is 3 min apart) in Hypergravity was explored via linear regression. Wilcoxon signed rank tests compared mean Baseline perception of each subject with their mean metrics from the Hypergravity segment and the first 12 min of the 1*g* Readaptation segment for both SVV and verbal tilt reports, and the signed rank statistic, *W*, is reported for each comparison. The Wilcoxon rank sum test is used to compare subgroups of subjects (i.e., those who received additional dynamic tilts vs. those who did not) in the 1*g* Readaptation segment, and the test statistic, *U*, is reported. Medians and interquartile ranges (IQRs) are reported. Statistical results are reported using α = 0.05, and *P‐*values are adjusted using a Bonferroni correction for multiple comparisons to the same set of Baseline measures.

#### 2.4.2 Computational modelling

**FIGURE 2:**
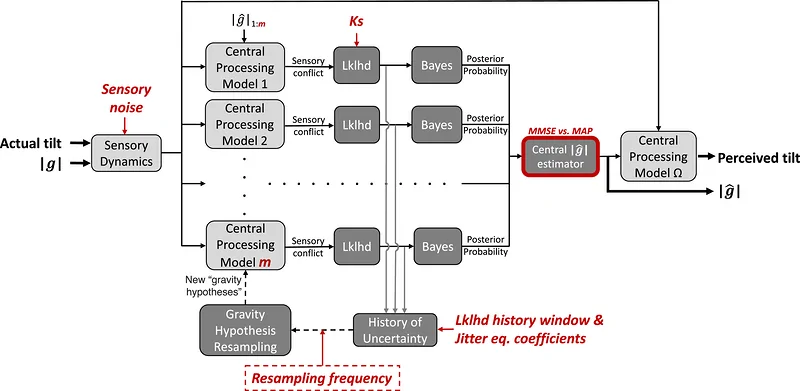
Model framework. The computational model framework discussed here was proposed by Kravets et al. (2021) and developed further by Kravets et al. (2022) and Allred et al. (2023). The inputs to the model are the actual tilt profile and the actual magnitude of gravity (|g|). It uses multiple (*m*) parallel versions of the observer model of spatial orientation perception (light grey sensory dynamics and central processing model blocks), each hypothesizing a different internally assumed magnitude of gravity, $|\hat{g}|$_1:_ *_m_*. The resulting sensory conflict leads to Bayesian probability computations and gravity hypothesis resampling, driving the internal ‘adaptation’ to the new gravity level. The main outputs of the model are the perceived tilt and internally assumed magnitude of gravity $(|\hat{g}|)\;$ over time. Parameters that can be used to tune the model to experimental data are highlighted in red.

The empirical data were used to fit the COMPASS computational model of vestibular adaptation proposed by Kravets et al. (2021) and developed further by Kravets et al. (2022) and Allred et al. (2023) (Figure 2). To summarize the computational framework briefly, it uses multiple (*m*), parallel versions of the well‐validated ‘observer’ model of spatial orientation perception (the sensory dynamics and central processing blocks in Figure 2) (Merfeld & Zupan, 2002; Merfeld, Young, Oman, et al., 1993; Merfeld, Young, Paige, et al., 1993; Vingerhoets et al., 2006, 2007). Each parallel ‘observer’ processes the same inputs (the actual roll tilt profile experienced in the actual magnitude of gravity ,|g|) and computes the resulting sensory conflicts based on different hypotheses of the internally assumed magnitude of gravity ($|\hat{g}|$ *_1:m_*), which are also referred to as ‘particles’ in the papers by Kravets et al. (2022) and Allred et al. (2023). The likelihoods of each $|\hat{g}|$ *_1:m_* given the sensory conflict signals generated by the parallel ‘central processing models’ are calculated. The hypotheses and their likelihoods are retained for a certain time window to measure sensory conflict likelihood accumulation used in the history of uncertainty in the hypotheses of gravity. This measure of uncertainty is used to calculate the jitter (or resampling distribution SD) when computing new gravity hypotheses such that higher uncertainty leads to a wider search window when resampling. The likelihoods are also used in the Bayesian inference step to determine the posterior probability of each hypothesis, and a central internally assumed magnitude of gravity is calculated using these posterior probabilities. This central internal assumption can be calculated using a variety of estimators, such as the minimum mean squared error (MMSE) estimate, which is effectively an average of the current hypotheses weighted by their posterior probabilities, or the maximum a priori (MAP) estimate, which is simply the hypothesis with the highest posterior probability. Here, results are reported using the MAP estimate. Finally, the central internally assumed magnitude of gravity ($|\hat{g}|$), in addition to the sensory dynamics, is fed into a final (Ω) central processing model to produce the perceived tilt associated with the actual tilt experienced over time.

This model framework involves several tuning parameters that need to be informed by experimental data (Figure 2, red text). Several of these parameters directly affect the gravity hypothesis resampling process: the number of parallel gravity hypotheses considered (*m*), the resampling frequency of these hypotheses, the temporal size (i.e., number of time steps) of the likelihood history window and the equation coefficients, *a* and *b*, used to calculate the resampling distribution jitter based on the uncertainty history. Here, jitter is calculated using an inverse logarithmic equation (jitter=a−b∗log[historyofthemaximumlikelihood]), whereas in the paper by Allred et al. (2023) a power equation was used to transform the hypothesis likelihoods to a jitter value. The logarithm and power equation produce qualitatively similar relationships between the history of maximum likelihoods and jitter, whereas the logarithmic relationship more appropriately scales the range of maximum likelihoods resulting from this set of tuning parameters to jitter values that do not expand the resampling window too quickly. Other tuning parameters include the noise covariance weighting parameter (*Ks*), the vestibular sensory noise magnitude and the choice of central estimator for $|\hat{g}|$ (MMSE vs. MAP). Further discussion of these tuning parameters can be found in the papers by Allred et al. (2023) and Kravets et al. (2022, 2021). Here, we tuned these parameters to best match the SVV experimental results from the day 1 session (i.e., the first adaptation to hypergravity). This tuning process was conducted via manual tuning and visual inspection to ensure that the model tilt perception predictions and empirical SVV reports corresponded.

As will be seen in the Results, subjects overestimated roll tilt while in the Hypergravity segment. To produce this effect of hypergravity in ‘observer’, Clark et al. (2015) proposed a weighting of the linear acceleration feedback gain, *Ka*, within observer to be −2 in the utricular plane and −4 perpendicular to the utricular plane (unitless). We adopted a similar approach here, but to match the amount of overestimation observed in our experiment we used −0.5 for the *Ka* component in the utricular plane.

## 3 RESULTS

### 3.1 Tilt overestimation results

#### 3.1.1 Tilt overestimation during the hypergravity segment

**FIGURE 3:**
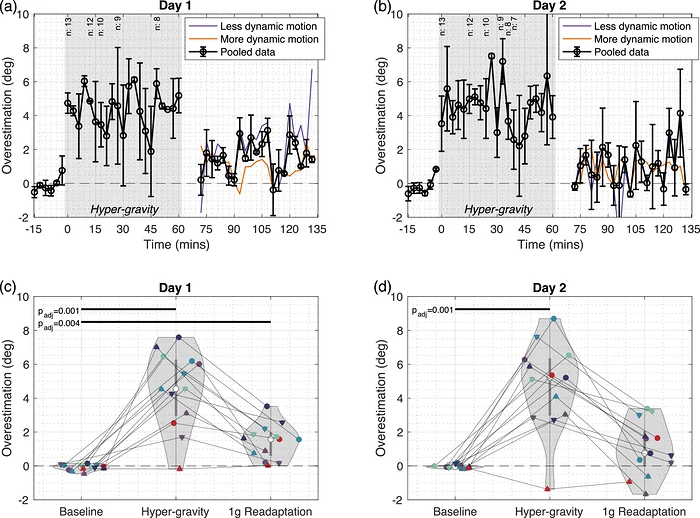
Tilt overestimation measured by subjective visual vertical (SVV). (a,b) The median tilt overestimation, with jackknife SE (Quenouille, 1956), measured by SVV tasks is shown for each time point on day 1 (a) and day 2 (b). Neither time (*P* = 0.911) nor day (*P* = 0.676) significantly impacted tilt overestimation in the simulated Hypergravity segment (grey background). The number of subjects pooled at each time point during hypergravity is indicated at the top of panels (a) and (b), and the 1*g* Readaptation reports for subjects are shown for the subsets of those who had minimal motion cueing (purple line), more dynamic motion cueing (red line) and the pooled set of all subjects (black line). (c) Using the set of SVV tasks in each experimental segment (e.g., Baseline) as replicates, there was significant overestimation of tilt in hypergravity relative to baseline (median, 4.6°; *W* = 1; *P*_adj_ = 0.001) and, surprisingly, also in the first 15 min of the 1*g* Readaptation segment (median, 1.6°; *P*_adj_ = 0.004) on day 1. (d) On day 2, there was significant evidence of overestimation in the Hypergravity segment (median, 5.2°; *W* = 1; *P*_adj_ = 0.001), but not in the 1*g* Readaptation segment (median, 0.7°; *W* = 15; *P*_adj_ = 0.066). In these panels, negative overestimation refers to underestimation, and 0° of overestimation indicates accurate tilt perception. Individual subjects are indicated with the same colour/marker on both days.

Tilt overestimation derived from SVV reports over the course of the experiment is shown in Figure 3. Here, positive values indicate overestimation of roll tilt, negative values indicate underestimation, and 0° indicates accurate perception. The time course of overestimation is shown in Figure 3a,b, in which the median values across subjects are shown at each time point. Subjects overestimated tilt during centrifugation, with a mean overestimation gain (i.e., $\mathrm{overestimation}/|\mathrm{til}t_{\mathrm{act}}|$) of 31% on day 1 (Figure 3a) and 33% on day 2 (Figure 3b). This overestimation appears consistent over the hour of centrifugation exposure, because linear regression indicated no evidence of adaptation (i.e., change in overestimation) during this time (coefficient estimate, −0.001 change in degrees of overestimation per minute of centrifugation; SE, 0.01; *t*‐statistic, −0.113; *P *= 0.911) and difference in the amount of overestimation across experiment days (coefficient estimate, 0.168 change in degrees of overestimation between days; SE, 0.398; *t*‐statistic, 0.422; *P *= 0.676).

After demonstrating the consistency of tilt overestimation over the course of the hour of centrifugation, we treated tilt perception trials during the same segment as replicates, and the mean SVV tilt reports by each subject across the different experimental segments on day 1 and day 2 are shown in Figure 3c,d, respectively. During the Hypergravity segment, subjects showed significant overestimation of head tilt relative to Baseline perception on both day 1 (median, 4.6°; IQR, 3.3°; *W* = 1; *P*_adj_ = 0.001, with Bonferroni correction for two comparisons with Baseline data on same day, i.e., Baseline vs. Hypergravity and Baseline vs. 1*g* Readaptation) and on day 2 (median, 5.2°; IQR, 3.4°; *W *= 1; *P*_adj_ = 0.001). Likewise, verbal reports also indicated overestimation of head tilt on day 1 (median,1.7°; IQR, 2.7°; *W *= 11; *P *= 0.013; *P*_adj_ = 0.026), but not on day 2 (median, 0.5°; IQR, 3.3°; *W* = 35; *P *= 0.497; *P*_adj_ = 1). Notably, the verbal reports indicated somewhat less overestimation than the SVV reports suggested.

Although the results above include all 13 subjects, we acknowledge that some of these subjects did not complete the entirety of the Hypergravity segment owing to excessive motion sickness (see Section 4.2). If we analyse the subset of subjects who did complete the entirety of both Hypergravity segments (*n* = 6, five males), we do find a significant change in overestimation over time during the Hypergravity segment (coefficient estimate, 0.031 change in degrees of overestimation per minute of centrifugation; SE, 0.013; *t*‐statistic, 2.420; *P* = 0.020). The positive coefficient suggests that this subgroup increased their overestimation over time. This corresponds to an increase in overestimation of 1.86° over the hour of centrifugation. These subjects showed no difference in the amount of overestimation across experiment days (coefficient estimate, 0.585 change in degrees of overestimation between days; SE, 0.470; *t*‐statistic, 1.246; *P* = 0.220). Although this finding is interesting to note, we pooled across subjects for further analysis owing to the small sample size of this subgroup.

#### 3.1.2 Tilt overestimation during the 1*g* Readaptation segment

If there had been adaptation in the Hypergravity segment (i.e., a change in overestimation over the 1 h of centrifugation), one might expect that there would be misperceptions of tilt (e.g., underestimation) during the 1*g* Readaptation segment. However, because we observed little evidence of adaptation to the 1.5*g* net force during the hour of centrifugation, minimal, if any, misperception was expected in the 12 min 1*g* Readaptation segment. Interestingly, the SVV results provided some evidence of overestimation in the 1*g* Readaptation segment on day 1 (median, 1.6°; IQR, 1.3°; *W* = 0; *P *= 0.002; *P*_adj_ = 0.004), although, with Bonferroni correction, this difference was not significant on day 2 (median, 0.7°; IQR, 2.1°; *W *= 15; *P *= 0.033; *P*_adj_ = 0.066). Considering only the subjects who completed the full hour of hypergravity exposure, this subset of subjects likewise showed evidence of overestimation during the 1*g* Readaptation segment relative to Baseline on day 1 (*n* = 8; median, 1.6°; IQR, 1.3°; *W* = 0; *P* = 0.008), but not on day 2 (*n* = 7; median, 0.4°; IQR, 1.4°; *W = *10; *P* = 0.578).

In contrast to the SVV tilt perception reports, verbal reports aligned more closely with the expected outcome during 1*g* Readaptation (i.e., because there was no evidence of adaptation in Hypergravity, tilt perception was expected to be roughly accurate when returning to 1*g*). Specifically, there was no significant evidence of overestimation of head tilt during the 1*g* Readaptation segment on either day 1 (median, 1.8°; IQR, 2.7°; *W *= 18; *P *= 0.057; *P*_adj_ = 0.114) or day 2 (median, −0.8°; IQR, 3.1°; *W *= 61; *P *= 0.305; *P*_adj_ = 0.610) via verbal tilt reports.

Given that we saw no change in tilt perception metrics over time in the Hypergravity segment (see Section 4.1.1), we did not expect the additional dynamic head tilts to affect tilt perception in the first 12 min of the 1*g* Readaptation period. A Wilcoxon rank sum test supports this hypothesis, indicating no significant difference in tilt overestimation during the first 12 min of readaptation between vestibular cueing subgroups on day 1 (*U* = 47; *P* = 0.836) or day 2 (*U* = 46; *P* = 0.731), hence data were pooled across subgroups for further analysis.

### 3.2 Motion sickness during centrifugation

Of the 13 subjects, eight completed the entire Hypergravity segment on day 1 and seven completed it on day 2. The number of subjects pooled at each time point is shown in Figure 3a,b. Subjects who concluded the Hypergravity segment early did so primarily owing to the onset of motion sickness, although, on day 2, one of the subjects experienced an HMD malfunction that led to the premature conclusion of the centrifugation segment. The median of the subjects’ maximum MISC scores was 2 (IQR, 2.75) on day 1 and 2 (IQR, 4.25) on day 2. Interestingly, there was some evidence that those who felt the most motion sickness (i.e., did not complete the full Hypergravity segment) showed a greater amount of overestimation upon initial exposure to centrifugation relative to those who completed the full Hypergravity segment. Further exploration of this relationship is necessary.

### 3.3 Computational model tuning outcomes

**FIGURE 4:**
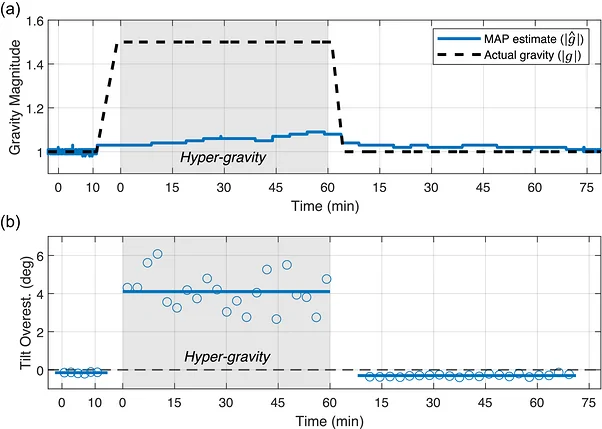
Simulation of the computational model using the exact experimental actual gravity and actual tilt profile. The actual experimental tilt and |g| profile was simulated using a tuned version of the COMPASS computational model, which outputs the internally assumed magnitude of gravity, $|\hat{g}|$, and generates perceived tilt from the final (Ω) central processing model (Figure 2). (a) Over the 1 h of hypergravity (grey background), the internally assumed magnitude of gravity (blue line) begins the early stages of adaptation to the actual gravity level (dashed black line). (b) The difference between the actual magnitude of gravity and the internally assumed magnitude of gravity leads to an overestimation of tilt during the Hypergravity segment, matching that observed experimentally. A slight amount of underestimation is predicted during the readaptation period following hypergravity exposure.

The SVV tilt perceptual report data from the Hypergravity segment on day 1 (first exposure to 1.5*g*) were used to fit the model to the time course of the experiment (Figure 4). Model simulations were performed with the exact time history of actual gravity (dashed black line in Figure 4a) and the sequence of actual head tilt angles (10°–20°, left and right head tilts). This yielded model predictions of perceived head tilt for the actual head tilt in each corresponding trial (Figure 4b), the mean of which was compared with the mean overestimation observed in empirical SVV reports. The model also predicted a time course of the MAP estimate of the internally assumed magnitude of gravity $\left(|\hat{g}|\right)$. Although this is an internal state that cannot be measured empirically, we can compare the internally assumed magnitude of gravity with the actual magnitude of gravity to quantify the time course of adaptation predicted by the model.

To match the empirical data appropriately, we settled upon the following set of tuning parameters. The covariance weighting constant, *Ks*, was set to one. Although this effectively removes the effect of *Ks*, increasing the value, as in the paper by Kravets et al. (2021), forces the spread of gravity hypotheses posterior probabilities to approach a uniform distribution and reduces the probability that new gravity hypotheses will be sampled from the region of the distribution closest to the actual gravity. The number of gravity hypotheses considered, *m*, was set equal to 10, which allows for exploration through resampling while limiting computational cost (i.e., the computer processing time required to run the simulations). The jitter equation coefficients, *a* and *b*, were set to 0.2 and 0.015, respectively, which determined the sensitivity of the jitter equation to changes in the uncertainty history. With these parameters set, the final tuning parameters, resampling frequency and jitter history window, can be used to adjust the resampling trajectory. For the simulation visualized in Figure 4, the resampling frequency was set to 3000 time steps (where a time step is 0.1 simulated seconds; i.e., 3000 time steps equals 5 simulated minutes), and the jitter history window was set to 5 × 10^5^ time steps (or ∼14 simulated hours). With these tuning parameters, the model is in only the early stages of adapting to the change in the magnitude of gravity by the end of the hour of hypergravity (Figure 4a). Owing to the difference between the actual magnitude of gravity (1.5*g*) and the internally assumed magnitude of gravity ($|\hat{g}|$) in the model (∼1*g*, up to 1.1*g*), the model predicts tilt overestimation during this period (Figure 4b). Notably, when the model was simulated through the 1*g* Readaptation segment, it did not predict overestimation of tilt using these tuning parameters and even predicted slight underestimation.

### 3.4 Tuned computational model simulations for longer‐duration adaptation to hypergravity

**FIGURE 5:**
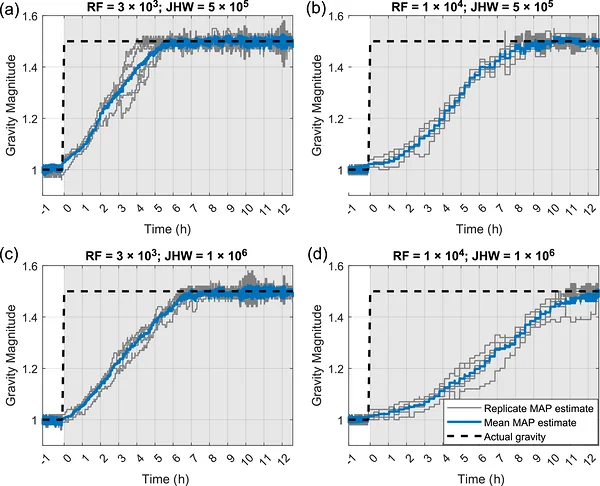
Longer‐duration simulation of the computational model with multiple sets of tuning parameters. Although the hour of centrifguation was not enough time to observe the full adaptation trajectory, the computational model can simulate the rest of a hypothetical adaptation profile, where the actual simulated gravity level is shown as the black dashed line in each panel. The stochastic nature of the model leads to slight variations between repeat simulations (grey lines), and the mean value of five simulations is shown for each set of parameters (blue line). (a) Extending the simulation time frame using identical tuning parameters to those in Figure 4 indicates that ∼5.5 h is needed for the internally assumed magnitude of gravity to match the actual gravity. (b–d) If motivated by future experimentation, the adaptation trajectory can be prolonged by: (b) increasing the resampling frequency (RF = 1 × 10^4^ time steps) while keeping the jitter history window constant; (c) increasing the jitter history window (JHW = 1 × 10^6^ time steps) while keeping the resampling frequency constant; or (d) increasing both tuning parameters (RF = 1 × 10^4^; JHW = 1 × 10^6^). In each of these simulations, *Ks* = 1, *m* = 10, *a* = 0.2 and *b* = 0.015.

With the constraints on the tuning parameters defined in the previous section, we next performed model simulations over longer time periods (≤12 h of 1.5*g* exposure), generating quantitative, testable hypotheses to inform and motivate future experiments regarding the time course of adaptation. Owing to the stochastic nature of the computational model, running a simulation with identical parameters multiple times can lead to slight variations in the adaptation trajectory, as shown by the underlying grey lines in each panel of Figure 5, which might correspond to intra‐individual variation given the same environmental cueing. Figure 5a depicts the full length of time predicted for adaptation using the same tuning parameters as the simulated experimental profile illustrated in Figure 4, predicting a need for ∼5.5 h in hypergravity for vestibular adaptation to the new gravity level. Figure 5 also illustrates how the model could be tuned to longer adaptation trajectories by increasing the resampling frequency or the jitter history window. Figure 5b,c shows the effect of increasing either of the parameters, and Figure 5d shows the effect of increasing both. With the resampling frequency set to ∼16.7 simulated minutes and the jitter history window considering 28 simulated hours of sensory conflict (Figure 5d), adaptation to the new gravity level would take ∼12 h (*Ks* = 1; *m *= 10; *a* = 0.2; *b *= 0.015; resampling frequency = 10,000 time steps or ∼16.7 simulated minutes; jitter history window = 1 × 10^6^ time steps or ∼28 simulated hours).

## 4 DISCUSSION

Using a hypergravity centrifugation protocol, we investigated the time course and severity of tilt overestimation over the first hour of exposure to altered gravity, and we used this knowledge to develop further an existing computational model of vestibular adaptation to changes in the magnitude of gravity. We extend the floor of the vestibular adaptation window to this length of time (with well‐controlled head movements and tilt perception reports intermittently throughout), which is important to know when considering space mission planning and crew safety. As anticipated, subjects overestimated head tilt upon entering a higher level of gravity (i.e., entering 1.5*g* when adapted to the normal 1.0*g*). With primarily vestibular sensory cues informing the internally assumed magnitude of gravity, this overestimation of tilt did not decrease over the course of an hour of exposure to the simulated gravity environment. These data help to constrain the parameters within the model, contributing to the development of a computational model quantifiably capturing the early time course of altered gravity neurovestibular adaptation driven by sensory conflict. Future studies will need to measure tilt perception over a time course of >1 h to bound these parameters fully. Several alternative sets of modelling parameters that can fit these data are presented, inspiring future experimental work to determine the full extent of gravity adaptation‐related misperceptions.

### 4.1 Tilt perception in altered gravity

Overall, the results presented here support previous evidence that initial exposure to a higher level of gravity than the adapted state leads to overestimation of head tilt (Clark et al., 2015). Clark and colleagues reported 17% overestimation in 1.5*g* and 35% overestimation in 2*g*, whereas the present subjects overestimated tilt by ∼30% in 1.5*g* [median overestimation across subjects was 4.6° (IQR, 3.3°), with an average tilt angle of ∼15°]. This difference in the observed magnitude of overestimation might result from methodological factors, in that Clark et al. (2015) incorporated whole‐body tilts (the head remained aligned with the body) and a subjective haptic horizontal measure of tilt perception, as opposed to the head‐on‐body tilts and SVV tasks used here. Additionally, the short radius of centrifugation used in the present study (∼1.5 m at the location of the head) might have led to an inflation of perceived tilt, because the net forces felt at the feet were greater than those felt at the head (the feet experienced a GIF equal to ∼2*g*, although not aligned with the longitudinal body axis). It is also plausible that random inter‐individual differences contributed to the greater overestimation quantified in the present study. The IQR of ±3.3° across subjects on the median overestimation of 4.6° in the present study corresponds to roughly 8%–49% overestimation in 1.5*g*. Reanalysing the raw data from Clark et al. (2015) on an individual subject basis found corresponding overestimation in 1.5*g* with an IQR of 11%–27%, which has substantial overlap with the present dataset, despite the methodological differences.

This emphasizes the high variability in individual responses to altered gravity quantified within the present study, both in tilt perception and in motion sickness. Although we did not see any effect of sex in these results, and the ages of subjects were generally homogeneous, we suspect that underlying vestibular sensitivity [i.e., quantified by vestibular perceptual thresholds (Allred & Clark, 2023; Kobel et al., 2021; Suri & Clark, 2020)] might play a role in vestibular responses to changes in the magnitude of gravity. Diaz‐Artiles and Karmali (2021) argued that vestibular thresholds, which vary between individuals, are related to vestibular sensory noise, and it has also been posited that neural noise saturation might modulate threshold discrimination predicted by Weber's law (Carriot et al., 2021). Although the intricacies of the relationship between threshold discrimination and neural noise are beyond the scope of the present work, computational model simulations suggest that sensory noise influences adaptation rates (Kravets et al., 2021). The data here are not sufficient to explore inter‐individual differences, but additional subject testing could shed some light on the underlying mechanisms and could inform tuning of the sensory noise parameter (Figure 2).

We also explored the effect of repeat exposure to the same altered gravity environment, with the intent of exploring the hypothesis that the CNS retains a memory of previous gravitational states. There is some evidence that astronauts with repeat exposure to microgravity readapt to Earth gravity more quickly than first‐time flyers (Paloski et al., 1999; Reschke et al., 1998; Schoenmaekers et al., 2022), which might be a result of learned states (Tjernström et al., 2002) or a restructuring of neural ‘learning’ circuitry, although these effects might also be confounded by cognitive/muscular strategies used to avoid motion sickness by repeat flyers (Clark, 2019). In the present study, we did not see any effect of ‘memory’ from exposure on the previous day, at least during the first hour of centrifugation. However, memory might influence the final time taken to adapt to altered gravity or other aspects of the adaptation trajectory not captured here. Furthermore, the impact of ‘memory’ from previous altered gravity exposures might take place only if there is substantial neurovestibular adaption to the first gravity exposure, which was not seen in the tilt perception data over the 1 h of centrifugation on day 1.

In addition to the first hour of exposure to hypergravity, we also captured tilt perception for 1 h in 1*g* following 1.5*g* exposure, with the intent to measure readaptation to 1*g*. The entire hour of readaptation data is visualized in Figure 3a,b, but we suspected that adaptation effects might subside rapidly, and thus analysed only the first 12 min of the 1*g* Readaptation segment. Although we hypothesized underestimation following centrifugation (Clark & Young, 2017; Galvan‐Garza et al., 2018; Meskers et al., 2021), this would be expected only if there was adaptation to hypergravity. Unexpectedly, there was some evidence of overestimation in 1*g* Readaptation reports on day 1, but post‐centrifugation tilt perceptions were similar to Baseline on day 2. Readaptation verbal reports on both days were not significantly different from Baseline, although these reports were confounded by the timing of data collection (i.e., the verbal reports were collected while the subject's head was returning to centre). We suspect that any additional readaptation effects might have subsided within the ∼12 min period between the final Hypergravity tilt perception trial and the first 1*g* Readaptation trial. Additionally, we hypothesize that if subjects had shown adaptation to hypergravity and thus underestimation of head tilt in the readaptation phase, more dynamic vestibular cueing would have increased the rate of readaptation.

Although perception of orientation in most everyday experiences integrates multiple sources of sensory information (Clark, 2019; Howard, 1982; Newman, 2009), isolating vestibular adaptation is useful for exploring adaptation to environments where visual cues might be absent or unreliable (e.g., owing to clouds/dust) and provides insight needed for future investigation of multisensory interactions. It is important to note, however, that incorporation of additional, reliable cues might speed up the adaptation process (Galvan‐Garza et al., 2018), and the CNS might reweight sensory information based on perceived reliability of the incoming sensory cues (Fetsch et al., 2012, 2009; Hupfeld et al., 2022).

### 4.2 Modelling vestibular adaptation

The sensory conflict‐driven Bayesian computations proposed by this model of vestibular adaptation to altered gravity are feasible processes that the CNS could execute. Cullen and collaborators (Jamali et al., 2019; Mackrous et al., 2019; Roy & Cullen, 2004) have quantified neural activity in animal models, particularly neurons in the vestibular nuclei and cerebellum, that exhibit behaviour analogous to the sensory conflict response characteristics. We are also not the first to hypothesize that the CNS uses Bayesian inference to revise internal model parameters and interpret vestibular information (Aitchison et al., 2021; Darlington et al., 2018; Körding & Wolpert, 2004; Laurens & Droulez, 2007). This includes the use of probabilistic models in sensorimotor learning (Körding & Wolpert, 2004) and the adjustment of stimulus–response times based on historical interactions with the stimulus (Darlington et al., 2018).

Bos and Bles (1998) have proposed an alternative model of adaptation to transitions in the magnitude of gravity, the subject vertical conflict (SVC) model. This model incorporates a low‐pass filter to assess both the magnitude and the direction of gravity by the CNS. The SVC model offers the advantage of dynamic estimation of the magnitude of gravity, unlike models rooted in the GIF resolution hypothesis, such as the observer model, which typically assume a constant internal gravity magnitude (Groen et al., 2022). However, internally estimated gravity magnitude in the SVC model adjusts over a time frame of tens of seconds. Based on the experimental evidence presented here (i.e., a 1 h lower bound of adaptation duration), the SVC model lacks the ability to represent the adaptation process accurately.

Alternatively, the COMPASS model of vestibular parameter adaptation can be tuned to fit a variety of different time lengths of adaptation based on experimental evidence. In the present model formulation, increasing the number of gravity hypotheses considered (*m*) tends to lower the posterior probability of even the most likely hypothesis, which can slow down the adaptation process while also increasing computational cost. The covariance weighting constant, *Ks*, is inversely proportional to the spread of the posterior distribution at a given time step. If *Ks* is too big, all the gravity hypotheses will have approximately the same posterior probability, regardless of their distance from the actual magnitude of gravity, leading to a more heavily stochastic adaptation process. The calculation of jitter, or gravity hypothesis resampling distribution SD, also greatly affects the adaptation process, because the entire adaptation process is driven by the spread of the sampled gravity hypotheses. A higher resampling frequency also slows down the adaptation process, because it means that there is more time between chances for the gravity hypotheses to converge on the actual magnitude of gravity. An extended jitter history window requires a larger period of sensory conflict build‐up before adaptation can begin. Many of these tuning parameters are closely intertwined, and further experimental evidence is needed to determine the effect that they might have in real‐life adaptation scenarios.

### 4.3 Limitations and future work

Although the main goal of the present study was to explore vestibular impairment during the early stages of adaptation to altered gravity, the full time line of the adaptation trajectory was not captured within the 1 h of centrifugation tested here. Extended (i.e., >1 h) durations of altered exposure with continuous human monitoring (measures of both vestibular impairment and sensory cueing) are necessary to capture the full course of adaptation. Exposure to microgravity during missions to the International Space Station (and the readaptation period after return to Earth) offers the chance to collect impairment data during the full course of adaptation to an altered gravity environment, but it is not feasible to control/monitor the sensory cues experienced after these gravity transitions fully. The complex and undocumented multisensory interactions complicate interpretation of any findings. Short‐radius centrifuges (such as the one used in the present study) and the high spin rate they require lead to subject discomfort, increased risk of Coriolis effects and motion sickness, limiting both the duration of hypergravity exposure and the dynamics of vestibular cueing that subjects can comfortably experience. Use of a long‐radius centrifuge could mitigate these limitations, but the feasibility and cost of these studies is important to consider. Finally, it is difficult to say with certainty whether adaptation to hypogravity (i.e., gravity levels <1*g*), particularly the unique and somewhat anomalous microgravity state (Merfeld, 2003), will follow the same trajectory as adaptation to hypergravity. These limitations highlight the need for (and possible difficulties with) future exploration of the gravity adaptation process.

## 5 CONCLUSIONS

In summary, here we present quantitative evidence of head tilt overestimation that lasts for ≥1 h after a transition to a simulated hypergravity environment. Although the vestibular impairment is expected to decay over time, the associated sensorimotor risks remain high during the first hour of exposure to a change in the gravity environment. We have used the collected data to inform the COMPASS model of neurovestibular adaptation to altered gravity and discuss a variety of sets of tuning parameters that can be chosen based on further evidence regarding adaptation trajectories. Although there is still much to learn about the vestibular adaptation process, the present study establishes a new lower bound for the time needed to adapt to a change in the magnitude of gravity, supporting the need for countermeasures or, at a minimum, careful mission planning during these first crucial moments in a new gravitational environment.

## AUTHOR CONTRIBUTIONS

This study was performed in the Bioastronautics Laboratory at the University of Colorado, Boulder. Victoria G. Kravets contributed to the conception of the work, acquired and analysed the data and drafted the manuscript. Torin K. Clark contributed to the conception of the work and interpretation of the data and critically revised the manuscript. Both authors approved the final version of the manuscript and agree to be accountable for all aspects of the work in ensuring that questions related to the accuracy or integrity of any part of the work are appropriately investigated and resolved. Both persons designated as authors qualify for authorship, and all those who qualify for authorship are listed.

## CONFLICT OF INTEREST

The authors declare no conflicts of interest.

## Data Availability

Data are available upon request to the corresponding author, Victoria G. Kravets.
